# Sex Differences in Determinants of Suicide Risk Preceding Psychiatric Admission: An Electronic Medical Record Study

**DOI:** 10.3389/fpsyt.2022.892225

**Published:** 2022-05-27

**Authors:** Robyn J. McQuaid, Katerina Nikolitch, Katie L. Vandeloo, Patricia Burhunduli, Jennifer L. Phillips

**Affiliations:** ^1^University of Ottawa Institute of Mental Health Research, Ottawa, ON, Canada; ^2^Department of Neuroscience, Carleton University, Ottawa, ON, Canada; ^3^School of Psychology, University of Ottawa, Ottawa, ON, Canada; ^4^Department of Psychiatry, University of Ottawa, Ottawa, ON, Canada; ^5^Department of Cellular and Molecular Medicine, University of Ottawa, Ottawa, ON, Canada; ^6^Department of Biochemistry, Microbiology and Immunology, University of Ottawa, Ottawa, ON, Canada

**Keywords:** electronic medical records, depression, inpatient psychiatry, psychosocial determinants, sex differences, suicidal ideation, suicide attempt

## Abstract

**Background:**

Individuals requiring inpatient psychiatric care represent a group at higher risk of progressing toward suicide attempt. Using electronic medical record (EMR) data collected from psychiatric inpatient admissions, the objective of this study was to identify sex differences in risk factors for suicide plans and/or attempts within the 30 days preceding hospital admission.

**Methods:**

Resident Assessment Instrument for Mental Health (RAI-MH) intake data were obtained for patients admitted to a Canadian tertiary-care hospital deemed a “threat or danger to self” during a 10-year period (2008–2018). Data was extracted for individuals categorized into three groups: non-suicidal (*N* = 568), presence of suicide plan (*N* = 178), and presence of suspected suicide attempt (*N* = 124) in the 30 days prior to hospital admission. Multivariate logistic regression models were used to examine determinants of suicide risk.

**Results:**

Across all models, diagnosis of depression was the strongest predictor of suicide plan and/or attempt (OR = 5.54, 95% CI = 3.71–8.27, *p* < 0.001). Comparing clinical symptoms between suicidal and non-suicidal groups at the time of admission, the largest effect sizes were found for hopelessness (*p* < 0.001, η^2^ = 0.11), and guilt or shame (*p* < 0.001, η^2^ = 0.09). Female sex was identified as a significant factor for elevated suicidal risk (OR = 1.56, 95% CI = 1.01–2.21, *p* = 0.01), thus we stratified the regression model by sex to identify specific risk factors for suicide plan and/or attempt for males and females. Among males, having no confidant (OR = 2.13, 95% CI = 1.19–3.80, *p* = 0.01), presence of recent stressors (OR = 1.95, 95% CI = 1.16–3.29, *p* = 0.01), and participation in social activities (OR = 1.67, 95% CI = 1.02–2.71, *p* = 0.04) were important predictors, while among females, younger age (OR = 0.96, 95% CI = 0.94–0.97, *p* < 0.001) increased odds of suicide plan and/or attempt.

**Conclusion:**

EMR-derived findings highlight different psychosocial and clinical determinants for males and females associated with suicide plan or attempt prior to psychiatric admission. Identifying precipitating factors that elevate imminent suicide risk may inform suicide prevention efforts for psychiatric inpatients.

## Introduction

Approximately 4,000 Canadians die by suicide every year (1) and for every suicide death there are an estimated 20 suicide attempts (2). Suicide disproportionately affects specific populations and groups (3–7), yet over 90% of individuals who die by suicide meet criteria for at least one psychiatric diagnosis and 60% meet criteria for a mood disorder (8, 9). Within psychiatric populations, at highest suicide risk are individuals with severe mental illness that necessitates inpatient care (10). Psychiatric inpatient admission often occurs at times of crisis when individuals might pose a threat of harm to themselves or others (11), however, identifying those at highest risk of suicide remains difficult. An additional challenge is to identify precipitating factors that may elevate imminent risk of suicide in vulnerable individuals.

Stressful life circumstances such as loss of income and residential instability have been associated with increased risk of suicidal ideation (thoughts of ending one’s life which may include a suicide plan) and suicide attempts (self-injurious behavior accompanied by an intent to die) (12–14). One’s social climate preceding the onset of suicidal behaviors is also important, and perceived loneliness uniquely predicts suicide attempt above and beyond depression (15). Furthermore, targeting hopelessness (a common symptom of major depressive disorder) appears to significantly reduce the depression-suicide link in both males and females (16). Identifying demographic and psychosocial factors present at the time of elevated suicide risk may lead to improved recognition of suicide vulnerability; unfortunately, existing literature on this subject remains sparse.

Nationally reported statistics on suicide rates highlight discrepancies between males and females. Females appear three-to-four times more likely to attempt suicide than males (17, 18), and are admitted to hospital following suicide attempt or self-injury more frequently (19, 20). However, males are three times more likely to die by suicide (17, 21). Such differences are often attributed to the fact that depression (a known risk factor for suicide) is more prevalent in females (22) and that males tend to use more lethal means during suicide attempt (23). Moreover, unique to females with a previous suicide attempt, severity of self-reported depressive symptoms predicted subsequent suicide attempts (24). The potential contributing role of psychosocial factors on sex-specific differences in suicidal behavior requires further study (25).

Recent research suggests that suicide risk evaluation could be improved through simultaneous use of electronic medical record (EMR) data and standardized risk assessment tools (26). Since 2008 in Ontario, Canada, all psychiatric inpatient service providers are required to assess patients at admission, discharge, and quarterly in the case of extended stays using the Resident Assessment Instrument for Mental Health (RAI-MH).^1^ The RAI-MH has well-established reliability and validity (27–29). RAI-MH admission data provides a snapshot of the demographic, psychosocial and clinical circumstances of individuals within an acute period prior to their hospital admission. Harnessing this data may permit identification of sex-specific factors present at the time of elevated suicide risk in individuals who had suicide plans and/or attempts in the period immediately preceding hospitalization.

The objectives of the current investigation were to: (1) identify factors associated with elevated suicide risk in the 30 days prior to admission; (2) identify factors that differentiate individuals with a suicide plan from those with a suspected suicide attempt; (3) determine whether suicide risk factors differ between males and females; and (4) compare mental health symptoms at the time of admission in relation to suicide risk. It was hypothesized that, in particular, psychosocial (i.e., recent life stressors, disrupted social relations), clinical (i.e., depression diagnosis), and socioeconomic indicators (i.e., income, residential stability) would be strongly linked to suicide risk. It was also predicted that female sex would be associated with elevated suicide risk compared to males. Moreover, it was hypothesized that clinical symptoms most closely aligned with depression would most strongly predict suicide risk.

## Materials and Methods

### Data Source

**FIGURE 1:**
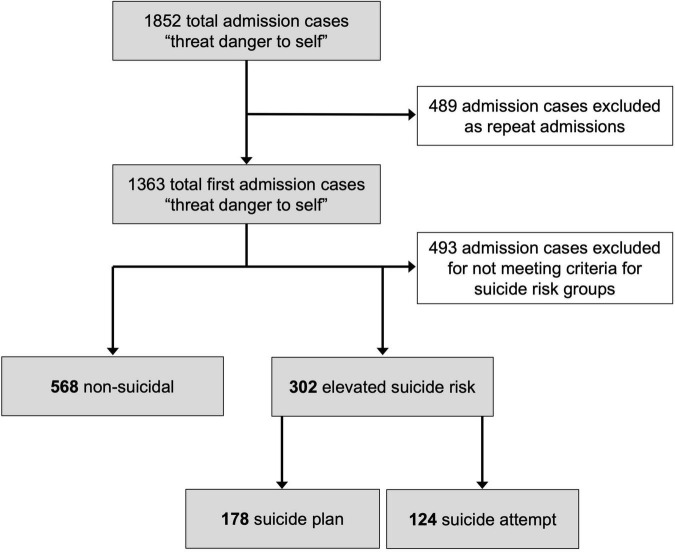
A flow diagram depicting the sample criteria and exclusions.

This study was a secondary analysis of EMR data recorded by clinicians at the point of psychiatric admission accessed from the RAI-MH database at the Royal Ottawa Mental Health Centre (ROMHC) in Ottawa, Canada. De-identified RAI-MH data were obtained for all admission cases in which “threat or danger to self” was coded as one of the reasons for admission over a 10-year period, from January 1, 2008 to December 31, 2017. Data from all ROMHC inpatient units (i.e., crisis, forensic, geriatric, mood, recovery, schizophrenia, and youth) were obtained. For individuals with multiple admissions over the 10-year period, only data from the first admission was included in the current study to avoid redundancy. From the resulting admission cases, each was categorized according to operationally defined groups based on pre-admission suicide risk level (see Figure 1). This study received approval from the ROMHC Research Ethics Board.

### Suicide Groups

Admission cases were organized into three groups based on self-injury related variables (Supplementary Table 1): (1) non-suicidal, operationally defined as admission case records where the data elements “considered performing a self-injurious act” and “most recent self-injurious act” were not endorsed for the previous 1-year period, there was no suicide plan in the 30 days prior to hospital admission, and no lifetime history of “fatal-intent self-injurious attempt,” (2) suicide plan, defined as endorsement of the presence of a suicide plan in the 30 days preceding admission but no “self-injurious attempt” within that time period, and (3) suspected suicide attempt, defined as coding of “most recent self-injurious attempt” within the 30 days prior to hospital admission, and positive endorsement of “intent of any self-injurious attempt was to kill himself/herself.” As the RAI-MH does not directly measure suicide attempts, these latter two variables were combined to reduce inclusion of individuals with recent engagement in non-suicidal self-injury in the suicide attempt group. Admission cases that did not meet inclusion criteria for any of the above-listed suicide groups were excluded. For example, admission cases with endorsement of suicidal ideation yet without plan or attempt, or with history of self-injurious attempt outside the timeframe examined (i.e., within past year but not past 30 days) were excluded. The resulting groups therefore comprised individuals deemed either non-suicidal, or at elevated suicide risk (recent suicide plan or suspected suicide attempt) from among available admission case data.

### Patient-Level Variables

The RAI-MH provides more than 300 individual data elements. Selecting plausible suicide risk variables based on previous literature and focusing on the time period immediately preceding admission, we extracted the following patient-level variables for each admission case (Supplementary Table 2): sex, age, marital status, education, sources of income, residential stability, stressors/life events, family roles, social relations, participation in social activities of long-standing interest, and psychiatric diagnosis. Certain variables were dichotomized to binary variables including education (“incomplete high school,” yes/no) and marital status (“single/unpartnered,” yes/no). For stressors, the RAI-MH lists individual variables for 16 adverse life events. Stressor categories were pooled into a single variable and binary coded as having occurred within the 30 days preceding admission (“stressor present,” yes/no). For family roles, positive endorsements of the RAI-MH variable “belief that relationship(s) with immediate family members is disturbed or dysfunctional” by the patient, family/friends, or both were pooled and the variable was binary coded (“belief present,” yes/no). Participation in social activities was binary coded according to occurrence during the 30 days preceding admission. Psychiatric diagnoses were determined by a physician during initial inpatient assessment using Diagnostic and Statistical Manual of Mental Disorders (DSM) criteria (30, 31). The RAI-MH also includes mental state indicators (scored as continuous variables) that report psychiatric symptom severity during the first 3 days of psychiatric hospitalization. Thirteen mental state indicators were selected for examination (Supplementary Table 3).

### Statistical Analysis

Data were analyzed with IBM SPSS Statistics (v26). A multivariate logistic regression model was conducted to investigate potential risk factors predicting the presence of suicide plan or attempt in the 30 days preceding hospital admission. For this analysis, individuals with either a suicide plan or suspected attempt were combined and compared to non-suicidal individuals. Additionally, a multivariate logistic regression model was conducted to differentiate individuals with a suicide plan from those with a suicide attempt (non-suicidal individuals were excluded from this analysis). To investigate the association between predictor variables and suicide risk specifically in males and females, the logistic regression models described above were stratified by sex. The examination of sex-stratified analyses may improve sensitivity to uncover effects that would not otherwise be found when the sexes are combined (32). Finally, multivariate analyses of variance (ANOVAs) were performed to compare mental state indicators for individuals deemed as non-suicidal vs. those at elevated suicide risk (with suicide plan or attempt).

## Results

### Descriptive Variables

A total of 1,852 admission cases were provided by the ROMHC (Figure 1). Of these, 489 admission cases were excluded as repeat admissions, and 493 first admission cases were excluded for not meeting criteria for inclusion in one of the suicide risk groups. The final retained dataset comprised admission cases from 870 individuals (*M_age_* = 48.3 years, *SE* = 0.75, range 15–97 years), of which 462 were females (53.1%) and 408 were males (46.9%). At the time of admission, 36.4% of individuals had not completed high school, and 60.3% reported residential instability. Moreover, 9.0% did not have a source of income; of those receiving income, only 6.3% was derived from employment. Remaining income sources included pension (45.9%), disability insurance (21.0%) and social assistance (11.0%). Demographic information is displayed according to groups in Table 1.

**TABLE 1:** Descriptives of non-suicidal and elevated suicide risk groups.

Characteristics	Non-suicidal (*N* = 568)	Suicide plan (*N* = 178)	Suicide attempt (*N* = 124)	Elevated suicide risk^a^ (*N* = 302)
Age, years (*M*, *SD*)	52.3 (± 0.90)	41.6 (± 1.46)	39.2 (± 1.75)	40.6 (± 1.23)
Male sex	57.2%	43.3%	48.4%	45.4%
Female sex	42.8%	56.7%	51.6%	54.6%
No source of income	5.6%	17.4%	12.1%	15.2%
Did not complete high school	35.1%	37.9%	40.3%	38.9%
Residential instability	62.1%	61.2%	50.8%	57.0%

*^a^The elevated suicide risk group combines the suicide plan and suicide attempt groups.*

Psychosocial and clinical factors deemed relevant to suicide plans and attempts were also assessed. At the time of admission, 18.4% of individuals reported having no confidant, 24.6% had disturbed or dysfunctional relationships with immediate family members, 24.9% of individuals experienced a stressor in the 30 days preceding admission, and 44.7% of individuals had not participated in social activities of long-standing interest in the 30 days preceding admission. Reported stressors were similar for males and females, with the most common being review hearing (7.0%), distress about the health of another person (6.6%), and conflict-laden relationships (6.1%). According to DSM diagnostic categories, included individuals had primary diagnoses of schizophrenia or other psychotic disorder (41.0%), depressive disorder (24.8%), neurocognitive disorder (14.6%), bipolar disorder (9.5%), substance-related or addictive disorder (3.5%), trauma or stressor-related disorder (2.8%), or other categories (each < 1.0%).

### Logistic Regression Model Assessing the Risk Factors of Elevated Suicide Risk

A logistic regression model comprising the main hypothesized variables was conducted to identify predictors of elevated suicide risk (presence of suicide plan or attempt) in the 30 days preceding admission. The overall model was significant [χ^2^(10) = 182.09, *p* < 0.001, *R*^2^ = 0.21]. As shown in Table 2, increased odds of elevated suicide risk was associated with younger age, female sex, disrupted family relationships, presence of a recent stressor, continued participation in social activities, having no confidant, and diagnosis of a depressive disorder. Sources of education, income, and residential instability were not significant in this model.

**TABLE 2:** Combined suicide risk model (logistic regression).

Model	β	SE	Significance	OR (95% CI)
Age	−0.03	0.005	<0.001	0.97 (0.97–0.98)
Sex	0.44	0.18	0.01	1.56 (1.01–2.21)
Low education	0.21	0.18	0.25	1.23 (0.86–1.76)
No Income	0.35	0.30	0.24	1.41 (0.79–2.54)
Residential instability	−0.18	0.18	0.31	0.84 (0.59–1.19)
Disrupted family relationships	0.43	0.20	0.03	1.54 (1.04–2.28)
Recent stressors	0.46	0.19	0.01	1.59 (1.10–2.31)
Participation in social activities	0.43	0.18	0.02	1.54 (1.09–2.19)
No confidant	0.44	0.22	0.04	1.55 (1.02–2.37)
Depression diagnosis	1.71	0.21	<0.001	5.54 (3.71–8.27)

### Sex-Based Analyses Assessing Risk Factors of Elevated Suicide Risk

The logistic regression model was stratified by sex to examine specific predictors of elevated suicide risk for males and females. This model was significant for males [χ^2^(9) = 48.89, *p* < 0.001, *R*^2^ = 0.11]. As shown in Table 3, the predictors that significantly increased the odds of suicide plan and/or attempt for males were the presence of recent stressors, continued participation in social activities, having no confidant, and depressive disorder diagnosis. All other variables including age, education, sources of income, residential instability, and disrupted relationships were non-significant. For females, the overall model was also significant [χ^2^(9) = 140.42, *p* < 0.001, *R*^2^ = 0.31]. Predictors associated with increased odds of suicide plan and/or attempt in females included younger age and diagnosis of a depressive disorder. None of the other predictors were significant, including education, sources of income, residential instability, disrupted relationships, recent stressors, social activities, and having no confidant (Table 3).

**TABLE 3:** Sex-stratified suicide risk models (logistic regressions).

Model	β	SE	Significance	OR (95% CI)
**Males**				
Age	−0.007	0.007	0.28	0.99 (0.98–1.01)
Low education	0.29	0.25	0.25	1.33 (0.82–2.17)
No income	0.48	0.40	0.23	1.61 (0.74–3.52)
Residential instability	−0.21	0.24	0.39	−0.81 (0.51–1.30)
Disrupted family relationships	0.36	0.30	0.23	1.44 (0.79–2.61)
Recent stressors	0.67	0.27	0.01	1.95 (1.16–3.29)
Participation in social activities	0.51	0.25	0.04	1.67 (1.02–2.71)
No confidant	0.76	0.30	0.01	2.13 (1.19–3.80)
Depression diagnosis	1.36	0.30	<0.001	3.89 (2.14–7.06)
**Females**				
Age	−0.5	0.007	<0.001	0.96 (0.94–0.97)
Low education	0.09	0.28	0.77	1.09 (0.62–1.90)
No Income	0.24	0.49	0.63	1.27 (0.49–3.31)
Residential instability	−0.10	0.28	0.73	−0.91 (0.52–1.58)
Disrupted family relationships	0.41	0.28	0.15	1.51 (0.86–2.63)
Recent stressors	0.29	0.28	0.31	1.33 (0.76–2.33)
Participation in social activities	0.30	0.27	0.26	1.35 (0.80–2.30)
No confidant	0.14	0.33	0.67	1.15 (0.60–2.19)
Depression diagnosis	1.95	0.29	<0.001	7.04 (3.97–12.47)

### Model Assessing Risk Factors for Suicide Plan vs. Attempts

The overall model assessing risk factors to differentially identify individuals who had a suicide plan vs. those who had an attempt in the 30 days preceding hospital admission was not significant [χ^2^(10) = 10.34, *p* = 0.28, *R*^2^ = 0.04].

### Mental State Indicators Associated With Elevated Suicide Risk

A multivariate ANOVA was conducted to distinguish mental state indicators in individuals with a recent suicide plan or attempt from those deemed non-suicidal in the period preceding admission. The overall model was significant [*Pillai’s Trace* = 0.20, *F*(14, 855) = 15.40, *p* < 0.001, η^2^ = 0.20]. As shown in Table 4, a number of indicators differed according to suicide risk, including: presence of sad, pained, worried facial expressions (*p* = 0.001, η^2^ = 0.01), making negative statements (*p* < 0.001, η^2^ = 0.07), self-deprecation (*p* < 0.001, η^2^ = 0.08), expressions of guilt or shame (*p* < 0.001, η^2^ = 0.09), statements of hopelessness (*p* < 0.001, η^2^ = 0.11), irritability (*p* < 0.001, η^2^ = 0.03), anxious complaints (*p* < 0.001, η^2^ = 0.03, η^2^ = 0.04), anhedonia (*p* < 0.001, η^2^ = 0.04), loss of interest (*p* < 0.001, η^2^ = 0.02), lack of motivation (*p* = 0.001, η^2^ = 0.01), and sleep problems (*p* = 0.026, η^2^ = 0.01). Two mental state indicators, episodes of panic (*p* < 0.10), and command hallucinations (*p* = 0.21), were not significant.

**TABLE 4:** Mental state indicators of non-suicidal and elevated suicide risk groups.

Clinical features	Non-suicidal (M ± SE)	Elevated suicide risk^a^ (M ± SE)
Facial expression**	1.77 ± 0.05	2.09 ± 0.07
Made negative statements***	1.09 ± 0.05	1.81 ± 0.07
Self-deprecation***	0.53 ± 0.05	1.20 ± 0.06
Guilt/shame***	0.44 ± 0.04	1.10 ± 0.06
Hopelessness***	0.54 ± 0.05	1.34 ± 0.06
Irritability***	1.47 ± 0.05	0.99 ± 0.07
Anxious complaints***	1.25 ± 0.05	1.80 ± 0.07
Episodes of panic	0.37 ± 0.03	0.29 ± 0.05
Anhedonia***	0.77 ± 0.05	1.31 ± 0.07
Loss of interest***	1.08 ± 0.05	1.48 ± 0.08
Lack of motivation**	1.21 ± 0.06	1.53 ± 0.08
Command hallucinations	0.29 ± 0.03	0.35 ± 0.07
Sleep problems*	0.96 ± 0.05	1.16 ± 0.07

*p < 0.05, **p < 0.01, ***p < 0.001.

*^a^The elevated suicide risk group combines the suicide plan and suicide attempt groups.*

### Sex-Based Analyses Assessing Mental State Indicators Related to Elevated Suicide Risk

When stratified by sex, multivariate ANOVAs were significant for males [*Pillai’s Trace* = 0.22, *F*(13, 448) = 9.86, *p* < 0.001, η^2^ = 0.22] and females [*Pillai’s Trace* = 0.18, *F*(13, 394) = 7.04, *p* < 0.001, η^2^ = 0.18]. While sex-stratified results remained similar to the above model with both males and females, in males alone, sleep problems (*p* = 0.23), episodes of panic (*p* = 0.39) and command hallucinations (*p* = 0.26) were not significant. For females, sleep problems (*p* = 0.09) and command hallucinations (*p* = 0.36) were not significant. In males, sad, pained, worried facial expression was significant (*p* < 0.01), while episodes of panic was significant in females (*p* < 0.001, η^2^ = 0.03).

### Mental State Indicators Associated With Suicide Plan vs. Attempts

Upon comparing mental state indicators in those with a suicide plan to those with an attempt prior to hospital admission, the overall model was not significant [*Pillai’s Trace* = 0.85, *F*(13, 288) = 1.15, *p* = 0.32, η^2^ = 0.05].

## Discussion

The current study harnessed EMR data from individuals admitted to a Canadian tertiary care psychiatric hospital deemed at risk to themselves. Data was compared for cases classified as non-suicidal or at elevated suicide risk (suicide plan or attempt) in the 30 days immediately preceding hospital admission. We report that among inpatients, depressive disorder diagnosis increases odds of elevated suicide risk, particularly for females. Further, inpatients with recent suicide plan or attempt reported more expressions of hopelessness, guilt and/or shame at admission. For males, having no confidant and experiencing recent stressors increased odds of suicide plan or attempt.

More than half of the sample experienced residential instability at the time of admission, which is consistent with research highlighting the strong connection between unstable housing and psychiatric disorders (33). Moreover, few individuals received income through employment, with the majority receiving a pension, social assistance, or disability insurance. These basic demographic data support literature linking indicators of low socioeconomic status (SES) as risk factors for mental illness (34–36). However, despite high rates of residential instability and low employment, SES indicators were not significant predicators of suicide plans or attempts, possibly due to the homogeneity of SES in this sample.

Diagnosis of depression was the strongest predictor of suicide plans or attempts across all models. Indeed, individuals with a primary depressive disorder diagnosis were at least five times more likely to have a suicide plan or attempt. This is in line with findings that 60% of individuals who die by suicide meet criteria for a mood disorder (8, 9). A diagnosis of depression is also a significant longitudinal predictor for suicidal ideation and attempts, although a recent meta-analysis suggests the effects of depression on suicide outcomes, including ideation, attempts and deaths, are not as strong as expected (37). Examining specific features of depression in relation to suicide outcomes might prove beneficial in identifying individual risk. In this regard, when considering hopelessness, the depression-suicide relationship is significantly reduced (16), and hopelessness is associated with suicidal ideation independent of depression severity (38). The current findings reveal that hopelessness had the largest effect size in relation to recent suicide plans or attempts across all mental state indicators, highlighting the potential importance of early identification of hopelessness. Expressions of guilt and shame had the next largest effect on suicide risk. In meta-analyses, guilt and shame have been associated with non-suicidal self-harm, however, few reports have examined these emotions in relation to suicide outcomes (39, 40). Thus, the current findings suggest that specific clinical symptoms related to depression such as hopelessness, guilt and shame may help identify elevated suicide risk in an inpatient psychiatric population. Finally, it may also be important to consider affective temperament alongside depression diagnosis as depressive, cyclothymic, anxious, and irritable temperaments have each been shown to be strongly associated with suicidal behavior (41). Although temperament was not assessed directly in our dataset, anxious complaints were more prevalent among those at elevated suicide risk, while irritability was higher in those deemed non-suicidal.

Across males and females, a depressive disorder diagnosis was a significant and strong predictor for suicide plans and attempts, increasing risk by four and seven times in males and females, respectively. Based on these data it was not surprising that female sex was a significant predictor of elevated suicide risk. Our data supports findings that females are more likely to attempt suicide compared to males (17, 18). Although males are historically more likely to die by suicide (17, 21), female rates of death by suicide have increased slightly from 1999 to 2019 in the United States (42, 43). Moreover, the proportion of individuals with past-year suicidal ideation who attempted suicide from 2008 to 2017 was significantly higher among females in the U.S. and among those aged 18–25 years (44). In Canada, females are more frequently admitted to hospital following suicide attempt or self-injury, and the recent increase in rate of hospitalization due to self-injury in young females in Canada is substantial (19, 20). From 2009 to 2014, the rate of intentional self-harm related hospitalizations increased by 110% in females and 35% for males aged 10–17 years (45). Similarly, from 2003 to 2017, increased rates of Ontario emergency department visits for self-harm and mental health were found for all youth aged 13–17 years, but especially among females (46). Herein, we report that both female sex and younger age were associated with elevated suicide risk, which support emerging trends.

For males, a number of psychosocial factors, including having no confidant, participating in social activities and presence of recent stressors were associated with increased risk of suicide plan or attempt. As stress is a longitudinal predictor of both suicidal ideation and attempts (47, 48), it was expected that stressful events would be identified as a significant risk factor. Males were also twice as likely to report recent suicide plans or attempts if they had no confidant. Due to masculinity norms, such as self-reliance, men are less likely to seek help for mental health issues (49). Although men express wanting to talk about their mental health and developing relationships to support these discussions (50), as in the current study, having no confidant would serve as a barrier. Surprisingly, although similar proportions of males and females reported having no confidant, it was not a significant risk factor for females. However, because depression was such a strong risk factor for females (OR = 7.04), it may have masked the effect of other variables. For males, participation in social activities of long-standing interest was positively associated with suicide plans or attempts, which was unexpected. However, the nature of these interactions is unknown; for example, the experience of unexpected, unsupportive social interactions is strongly related to poor mental health (51). It is also possible that males with suicidal ideation try to disguise their worsening mental state by engaging in their usual associations and activities. This is consistent with reports that males are more likely than females to self-stigmatize depression and suicidal thoughts and behaviors (52). In any case, our results show that psychosocial factors such as stressful experiences and social relationships are particularly important in screening for elevated suicide risk in males. Moreover, targeted social interventions such as offering social skills training and social support, providing opportunities for positive social engagement, and challenging maladaptive social cognition (53) may prove beneficial for suicide prevention efforts (54).

This study used EMR data to examine demographic and psychosocial characteristics of individuals at elevated suicide risk prior to psychiatric hospital admission. Strengths of this study included the large, representative, transdiagnostic sample of psychiatric inpatients and careful examination of sex-specific findings. The study also had a number of limitations. First, the RAI-MH does not directly measure suicide attempts, thus, suspected suicide attempts were derived from two separate variables. Ultimately, it would have been preferable to have suicide attempts confirmed and coded in the data. Second, the study comprised data from a single tertiary care psychiatric hospital with no emergency department. While this could also be viewed as a strength, as this investigation was targeted toward inpatients with severe mental illness who are deemed at high risk of suicide, it is also a limitation as there could be delays between first presenting at another hospital with an emergency department and admission to the ROMHC. Third, data were obtained only for individuals deemed a “threat or danger to self” at admission. Those we classified as non-suicidal may have had greater issues with self-care or specific psychiatric symptoms that posed a threat to their wellbeing, rather than being at risk of suicide at admission. Fourth, the extracted data corresponded only to a single time-point (admission) and was largely collected retrospectively. Fifth, several variables were dichotomized for ease of interpretation, yet this action may have reduced statistical power. Finally, while a number of demographic variables were included, data on race, ethnicity and gender identity are not fully defined or collected in the RAI-MH. Therefore, while we recognize that suicide disproportionately affects specific and/or marginalized populations (3–7), we were not able to examine these factors in our models.

Predicting suicide on an individual level remains exceptionally challenging. Ultimately, we could not tease apart those who transition from suicide plan to attempt. To help differentiate the transition from suicide thoughts to behaviors, alternative approaches including machine learning (55) may prove valuable in predicting suicide risk beyond hypothesis-driven approaches. Despite this, the current findings highlight risk factors associated with progression toward forming a suicide plan or attempt. Thus, identifying individuals at highest risk of suicide, particularly in a high-risk population, is an important first step toward suicide prevention efforts. Together, our findings suggest that depression is a very strong risk factor for suicide plans and attempts, particularly for younger females. Finally, this data reveals that having no confidant doubles the risk of suicide plans or attempts in males, suggesting that interventions to provide social support to men in distress and increase help seeking behaviors for mental health could prove beneficial for suicide prevention.

## Data Availability Statement

The data analyzed in this study is subject to the following licenses/restrictions: The current investigation is a secondary analysis of electronic medical record data from the Royal Ottawa Mental Health Centre. Thus, this data is not owned by the authors and cannot be made publicly available. However, this and other Ontario hospital data collected through the Ontario Mental Health Reporting System (OMHRS) can be requested from the Canadian Institutes of Health Information. Requests to access these datasets should be directed to https://www.cihi.ca/en.

## Ethics Statement

The studies involving human participants were reviewed and approved by the Research Ethics Board of the Royal Ottawa Mental Health Centre. Written informed consent for participation was not required for this study in accordance with the national legislation and the institutional requirements.

## Author Contributions

JP and KN were responsible of the overall research idea and acquisition of data. JP, KV, and PB contributed to data extraction and management. RM performed the statistical analyses. JP, RM, and KN led scientific interpretation of the data. RM wrote the first manuscript draft while JP and KV made substantial manuscript revisions. All authors critically reviewed, edited, and approved the final manuscript.

## Conflict of Interest

The authors declare that the research was conducted in the absence of any commercial or financial relationships that could be construed as a potential conflict of interest.

## Publisher’s Note

All claims expressed in this article are solely those of the authors and do not necessarily represent those of their affiliated organizations, or those of the publisher, the editors and the reviewers. Any product that may be evaluated in this article, or claim that may be made by its manufacturer, is not guaranteed or endorsed by the publisher.

## Footnotes

1
http://www.interrai.org/
