# Supplementary material for: Sex Differences in Determinants of Suicide Risk Preceding Psychiatric Admission: An Electronic Medical Record Study
Source: Front Psychiatry. 2022 May 27;13:892225. doi: 10.3389/fpsyt.2022.892225 (PMC9196272; doi:10.3389/fpsyt.2022.892225)
Supplement: Supplementary file 1 [file Data_Sheet_1.PDF]

Supplemental Materials

Sex differences in determinants of suicide risk preceding psychiatric admission: An electronic medical record study (McQuaid et al.)

Table S1. Classifications and definitions of suicide groups from RAI-MH data elements.

RAI-MH Section	RAI-MH Data Element	Suicide Group	Operational Definition
D.1 Self Injury	Code for the most recent instance.<br>a. Most recent self-injurious attempt<br>0. Never<br>1. More than 1 year ago<br>2. 31 days to 1 year ago<br>3. 8 to 30 days ago<br>4. 4 to 7 days ago<br>5. In last 3 days<br><br>b. Intent of any self-injurious attempt was to kill himself/herself<br>0. No<br>1. Yes<br>8. No attempt<br><br>c. Considered performing a self-injurious act<br>0. Never<br>1. More than 1 year ago<br>2. 31 days to 1 year ago<br>3. 8 to 30 days ago<br>4. 4 to 7 days ago<br>5. In last 3 days<br><br>db. Suicide plan—in last 30 days, formulated a scheme to end own life<br>0. No<br>1. Yes	Non-suicidal<br><br>Suicide Plan<br><br>Suspected Suicide Attempt	a = 0-1<br>b = 0, 8<br>c = 0-1<br>db = 0<br><br>a = 0-2<br>db = 1<br><br>a = 3-5<br>b = 1

Abbreviation: Resident Assessment Instrument for Mental Health (RAI-MH) (1).

Table S2. Derivation and coding of suicide risk factors from RAI-MH data elements.

Risk Factor	RAI-MH Section	RAI-MH Data Element	Patient-level Variable Coding
Sex	BB.1 Sex	M. Male<br>F. Female<br>O. Other	male = M<br>female = F
Age	Calculated by data extraction team from BB.2 Birthdate and CC.1 Date stay began	Year/Month/Day	age in years
Single/unpartnered marital status	BB.3 Marital Status	1. Never married<br>2. Married<br>3. Partner/significant other<br>4. Widowed<br>5. Separated<br>6. Divorced	"single/unpartnered", yes/no<br>yes = 1, 4-6<br>no = 2-3
Less than high school education	BB.5 Education	1. No schooling<br>2. 8 grades or less<br>3. 9–11 grades<br>4. High school<br>5. Technical or trade school<br>6. Some college/university<br>7. Diploma/bachelor's degree<br>8. Graduate degree<br>9. Unknown	"incomplete high school education", yes/no<br>yes = 1-3<br>no = 4-8
Having no source(s) of income	BB.6 Sources of Income	0. No 1. Yes<br>a. Employment<br>b. Employment insurance<br>c. Pension (CPP, ODSP, etc.)<br>d. Social assistance<br>e. Disability insurance<br>f. Other<br>g. No income	"no income", yes/no<br>yes = g<br>no = a-f
Residential instability	CC.5 Residential Stability	Prior to admission, most recent residence was temporary (e.g. shelter).<br>0. No 1. Yes	"residential instability", yes/no<br>yes = 1<br>no = 0
Experienced stressor(s) in past 30 days	J.1 Life Events	Code for the most recent time of the event.<br>0. Never<br>1. More than 1 year ago<br>2. 31 days to 1 year<br>3. 8 to 30 days<br>4. 4 to 7 days<br>5. In last 3 days<br>a. Serious accident or physical impairment	"stressor present in 30 days preceding admission", yes/no<br>yes = 3-5 and a-p<br>no = 0-2

		b. Distress about health of another person<br>c. Death of close family member or friend<br>d. Child custody issues, birth or adoption of child<br>e. Conflict-laden or severed relationship, including divorce<br>f. Failed or dropped out of education program<br>g. Major loss of income or serious economic hardship<br>due to poverty<br>h. Review hearing, e.g. forensic, certification, capacity hearing<br>i. Immigration, including refugee status<br>j. Lived in war zone or area of violent conflict (includes combatants and civilians)<br>k. Witness to severe accident, disaster, act of terrorism,<br>violence or abuse<br>l. Victim of crime (e.g., robbery). Exclude assault.<br>m. Victim of sexual assault/abuse<br>n. Victim of physical assault/abuse<br>o. Victim of emotional abuse<br>p. Parental abuse of alcohol or drugs	
Disturbed or dysfunctional relationships with immediate family members	O.1 Family Roles	Belief that relationship(s) with immediate family members<br>is disturbed or dysfunctional.<br>0. Belief not present<br>1. Only person believes<br>2. Family/friends/others believe<br>3. Both person and family/friends/others believe	"belief that relationship(s) with immediate family members<br>is disturbed or dysfunctional" present, yes/no<br>yes = 1-3<br>no = 0

Having no confidant	O.2 Social Relations and Interpersonal Conflict	a. Reports having no confidant.<br>0. No 1. Yes	“reports having no confidant”, yes/no<br>yes = 1<br>no = 0
Participation in social activities in past 30 days	O.6 Social Relationships	a. Participation in social activities of long-standing interest<br>0. Occurred within the last 3 days<br>1. Occurred within the last week<br>2. Occurred within the last month<br>3. Last occurred more than 1 month ago	“participation in social activities of long standing interest”, yes/no<br>yes = 0-2<br>no = 3
Psychiatric diagnosis	Q.1 DSM-5 Diagnostic Category*	Code “1” for the most important diagnosis<br>a. Neurodevelopmental disorders<br>b. Schizophrenia spectrum and other psychotic disorders<br>c. Bipolar and related disorders<br>d. Depressive disorders<br>e. Anxiety disorders<br>f. Obsessive-compulsive and related disorders<br>g. Trauma- and stressor-related disorders<br>h. Dissociative disorders<br>i. Somatic symptoms and related disorders<br>j. Feeding and eating disorders<br>k. Eliminations disorders<br>l. Sleep-wake disorders<br>m. Sexual dysfunctions<br>n. Gender dysphoria<br>o. Disruptive, impulse-control and conduct disorders<br>p. Substance-related and addictive disorders<br>q. Neurocognitive disorders<br>r. Personality disorders<br>s. Paraphilic disorders<br>t. Other mental disorders<br>u. Medication-induced movement disorders and	“primary psychiatric diagnostic category”, yes/no<br>yes = 1<br>no = 0

		other adverse effects of medication<br>v. Not applicable	

Abbreviations: Resident Assessment Instrument for Mental Health (RAI-MH) (1); Diagnostic and Statistical Manual of Mental Disorders, 5th Edition (DSM-5) (2)

*For early admission cases, psychiatric diagnosis was characterized as per criteria in the Diagnostic and Statistical Manual of Mental Disorders, 4th Edition, Text Revision (DSM-IV-TR) (3). In such cases, DSM-IV-TR psychiatric diagnoses were re-classified into appropriate DSM-5 diagnostic categories.

Table S3. Name and description of mental state indicators examined.

RAI-MH Data Element	Mental state indicator	Description
B1a	Facial expression	Sad, pained, worried facial expression (e.g. furrowed brow)
B1d	Made negative statements	Made negative statements (e.g. "Nothing matters," "I would rather be dead," "Let me die," "What's the use?"); regrets having lived so long.
B1e	Self-deprecation	Self-deprecation (e.g. "I am nothing," "I am no use to anyone.")
B1f	Guilt/Shame	Expressions of guilt or shame (e.g. "I've done something awful," "This is all my fault," "I am a terrible person.")
B1g	Hopelessness	Statements of hopelessness (e.g. "There's no hope for the future," "Nothing's going to change for the better.")
B1j	Irritability	Marked increase in being short-tempered or easily upset
B1o	Anxious complaints	Repetitive anxious complaints (non-health-related) (e.g. persistently seeks attention/reassurance)
B1t	Episodes of panic	Unexpectedly overwhelmed by sense of panic
B1v	Command hallucinations	Hallucinations directing the person to do something or to act in a particular manner (e.g. to harm self or others)
B1y	Anhedonia	Statements that indicate a general lack of pleasure in life (e.g. "I don't enjoy anything anymore.")
B1z	Loss of interest	Withdrawal from activities of interest or from longstanding social relations (e.g. no interest in longstanding activities or being with family/friends)
B1aa	Lack of motivation	Absence of spontaneous goal-directed activities
B1gg	Sleep problems	Any sleep problems present: difficulty falling asleep, restless or non-restful sleep, interrupted sleep (including awakening earlier than desired), too much sleep)

Abbreviation: Resident Assessment Instrument for Mental Health (RAI-MH) (1)

References

1. Hirdes JP, Smith TF, Rabinowitz T, Yamauchi K, Pérez E, Telegdi NC, et al. The Resident Assessment Instrument–Mental Health (RAI-MH): inter-rater reliability and convergent validity. J Behav Health Serv Res. 2002;29(4):419-432. doi: 10.1007/BF02287348

2. American Psychiatric Association: Diagnostic and Statistical Manual of Mental Disorders, 5th ed (DSM-V). Washington, DC, American Psychiatric Association, 2013.

3. American Psychiatric Association: Diagnostic and Statistical Manual of Mental Disorders, 4th ed, text revision (DSM-IV-TR). Washington, DC, American Psychiatric Association, 2000.
